# Evaluation and Use of *In-Silico* Structure-Based Epitope Prediction with Foot-and-Mouth Disease Virus

**DOI:** 10.1371/journal.pone.0061122

**Published:** 2013-05-07

**Authors:** Daryl W. Borley, Mana Mahapatra, David J. Paton, Robert M. Esnouf, David I. Stuart, Elizabeth E. Fry

**Affiliations:** 1 The Pirbright Institute, Pirbright, United Kingdom; 2 Division of Structural Biology, University of Oxford, The Henry Wellcome Building for Genomic Medicine, Headington, Oxford, United Kingdom; 3 Diamond Light Source, Harwell Science and Innovation Campus, Didcot, United Kingdom; CSIR-Institute of Microbial Technology, India

## Abstract

Understanding virus antigenicity is of fundamental importance for the development of better, more cross-reactive vaccines. However, as far as we are aware, no systematic work has yet been conducted using the 3D structure of a virus to identify novel epitopes. Therefore we have extended several existing structural prediction algorithms to build a method for identifying epitopes on the appropriate outer surface of intact virus capsids (which are structurally different from globular proteins in both shape and arrangement of multiple repeated elements) and applied it here as a proof of principle concept to the capsid of foot-and-mouth disease virus (FMDV). We have analysed how reliably several freely available structure-based B cell epitope prediction programs can identify already known viral epitopes of FMDV in the context of the viral capsid. To do this we constructed a simple objective metric to measure the sensitivity and discrimination of such algorithms. After optimising the parameters for five methods using an independent training set we used this measure to evaluate the methods. Individually any one algorithm performed rather poorly (three performing better than the other two) suggesting that there may be value in developing virus-specific software. Taking a very conservative approach requiring a consensus between all three top methods predicts a number of previously described antigenic residues as potential epitopes on more than one serotype of FMDV, consistent with experimental results. The consensus results identified novel residues as potential epitopes on more than one serotype. These include residues 190–192 of VP2 (not previously determined to be antigenic), residues 69–71 and 193–197 of VP3 spanning the pentamer-pentamer interface, and another region incorporating residues 83, 84 and 169–174 of VP1 (all only previously experimentally defined on serotype A). The computer programs needed to create a semi-automated procedure for carrying out this epitope prediction method are presented.

## Introduction

Foot-and-mouth disease (FMD) is an economically important disease that predominantly affects cloven-hoofed mammals, with the primary hosts being cattle, sheep, pigs and goats. The disease is currently endemic in Africa, South America and Asia. Recent outbreaks of the disease in the Far East [1] and Eastern Europe [2] demonstrate the ability of FMD to disseminate into areas previously free from disease with major economic impact. Although FMD does not have a high mortality rate in adult animals, it reduces the productivity of infected herds. It also seriously damages the economies of enzootic countries by impeding exports of livestock and livestock products.

Foot-and-mouth disease viruses (FMDV) are single stranded, positive sense RNA viruses belonging to the family *Picornaviridae* and are currently classified into 7 serotypes: A, O, C, SAT (South African territories) 1–3 and Asia-1. These serotypes share an approximate 86% amino acid identity to each other [3], however some of the capsid proteins exhibit more variation, notably VP1 which varies by 30–50% between serotypes [4]. This variation has impeded the development of vaccines that can provide cross protection both inter and intra-serotypically [5]. A vaccine providing broader antigenic coverage would be a valuable tool for the control of FMDV.

**Figure 1:**
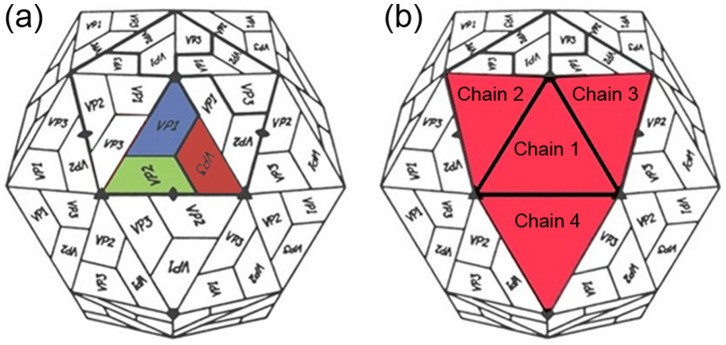
Building the multimer. (a) Schematic depiction of the foot and mouth disease icosahedral capsid with the individual virion peptides labelled. A viral subunit is coloured as per the standard colouring sytem; VP1 is blue, VP2 is in green and VP3 is red. (b) Schematic depiction of the of the same structure as (a) with the four subunits selected to make the multimer labelled as chains 1–4 (highlighted in red). These four protomers form the multimeric structure that was used in the analysis.

FMDV is a small icosahedral virus 30 nm in diameter, comprising 60 copies of each of the 4 structural proteins VP1-4 (see Figure 1(a)). VP1, 2 and 3 constitute the surface of the virus and are composed of 8 anti-parallel β strands linked by loops to form a β barrel. VP4 is much smaller, internal and has little secondary structure [6]. The highly mobile VP1 G-H loop protrudes from the surface of the virus and contains the arginine-glycine-aspartic acid (RGD) motif responsible for attachment to host integrins [7]. This loop is also antigenic on all serotypes of FMDV. Due to its disorganised (flexible) nature this loop is absent from many of the crystallographic structures reported, although it has been visualised when stabilised by chemical reduction [8] or bound to monoclonal antibody [9].

The role of antibody as the principal component of the immune response to FMDV is well established [10] and several immunodominant neutralising antigenic sites have been described on the surface exposed proteins, VP1-3. However, it has been shown that immunoglobulin specific protection can be achieved in the absence of a response to these epitopes [11], demonstrating the presence of other as yet unidentified epitopes. Traditional methods for identifying epitopes have relied on the generation of neutralising monoclonal antibody (mAb) escape mutants or peptide scanning techniques. These methods are time consuming, expensive and have generally utilised murine mAb’s. Novel methods have recently been developed to correlate amino acid changes on the capsid and serological changes for multiple virus pairs [12], [13]. Again, these methods require a large dataset and rely on amino acid variation within the capsid sequences to identify epitopes, thereby eliminating any possibility of identifying epitopes that are completely conserved. Such epitopes are particularly interesting as a potential route to more broadly reactive vaccines [14].

Many computational algorithms that try to predict B cell epitopes have been based on the analysis of sequence data alone. The first method described [15] used hydrophillicity scales of the amino acids averaged across the sequence tested. Subsequently, other methods have been described that utilize various sequence scoring algorithms [16], [17]. An evaluation of the predictive power of these programs suggested that none could reliably determine epitopes [18], which is perhaps not a surprise as these programs would have no way of determining solvent exposure. However, it has been suggested that using a consensus of the results from B cell epitope prediction programs may increase confidence in the predictions made [19]. Additionally, these programs are limited to predicting continuous epitopes, which are believed to comprise only 10% of the total number of available epitopes [20].

To identify discontinuous epitopes the three dimensional structure must be included in the analysis [20] since this determines which areas of the sequence come into close enough proximity to form a discontinuous epitope. Recently, several web based servers (see methods section for selected list) have become available that utilize both structural and sequence information to identify conformational epitopes on the surface of a protein. As these servers use a single set of structural and sequence data they can, in principle, detect epitopes that are completely conserved in sequence both within, and between serotypes. The algorithms assign each residue a score based on the likelihood of it forming part of a conformational epitope. The performance of these algorithms has been tested using databases of known epitopes from available structural data (*i.e.* the Epitome database- http://www.rostlab.org/services/epitome/). For additional information on the individual programs see references [21]–[25]. Although all of these programs have reported higher levels of success in prediction of epitopes than those that relied on sequence information alone, there has been no work conducted looking specifically at the performance of these programs using 3D structure information that is available for virus capsids. In order to fully understand virus antigenicity it must be examined at the level of the capsid so that interactions between the repeated protomeric subunits are accounted for.

The aim of our work is to examine the potential power of current structure-based approaches to predict unidentified Picornavirus epitopes using an appropriate subset of the icosahedral capsid which contains a central protomeric unit augmented by neighbouring structures sufficient to ensure that epitopes spanning symmetry related protomers can be identified (Figure 1). There is potential utility in such an approach, since it could provide a much more rapid, cheap and easy means of predicting viral epitopes conserved across several serotypes compared to the expensive and time consuming serological methods. We firstly define objective measures for sensitivity and specificity and then evaluate the best parameters for a selection of programs using an independent training set of known epitopes/structures from related picornaviruses (Poliovirus and Rhinovirus) before evaluating their performance in terms of their ability to identify already known FMDV epitopes for all serotypes for which a structure is available. On the basis of these results we use a consensus of the outputs of the more reliable programs to predict further antigenic regions on the capsid of FMDV. The results, which are the first to look at virus structures specifically in this context, demonstrate that there are severe limitations with the predictive power of each of the programs evaluated; however the predictions made using the consensus data are of sufficient interest to justify further experimental investigations.

## Methods

### Program Selection

Five freely accessible web-based B cell epitope prediction servers were selected for evaluation. These were BEPro [22] Discotope [23], Ellipro [25], Epitopia [21] and Seppa [24]. These are believed to encompass the most recent programs available (at the time of the evaluation), with the exclusion of the EPCES and EPSVR servers [26], for which the size of the Protein data bank (PDB) files for the multimeric structures being used was prohibitive.

Generally, for each program, a single value is assigned to each residue predicting its likelihood of being an epitope. However, for Epitopia two values are included in the output for each residue, one being an immunogenicity score and the second being a probability score. For the purposes of this analysis the probability score was used to determine the optimal threshold for identification of Picornavirus epitopes. Since the two values are strongly correlated this should not impact on the results.

### Structure Selection

Two Picornavirus structures were selected for program optimisation; Poliovirus P1 Mahoney strain (PDB code 1HXS- [27]) and Rhinovirus 14 (4RHV- [28]). These were chosen as they are the most well characterised Picornaviruses in terms of identified epitope residues. For FMDV, a structure was selected for each serotype (where available) for program evaluation. The list of structures used was as follows: Serotype C-S8c1 (1FMD- [29]), A1061 (1ZBE: [30]), O (O_1_ Kaufbeuren- [31]), SAT-1 (2WZR- [13]). An additional structure for serotype O (O_1_ Kaufbeuren- data not published) with the G-H loop present in a reduced conformation was also included in order to evaluate the impact of the presence of the G-H loop.

### Sequence and Conformational Comparison of Structures

Poliovirus and Rhinovirus are both members of the enterovirus genera of the Picornaviruses. The sequences of the two virus structures examined here share a 50% identity to each other while the structures have an average root-mean-square distance (RMSD) between 765 structurally equivalent C alpha atoms of 0.96 Å (calculated with SHP [32]). The sequences of these two enteroviruses have 22–24% identity with the sequences of the four FMD viruses we use in the subsequent evaluation (calculated in MEGA 5 [32] using a p-dist matrix and multiplying by 100 - used for all Identity calculations). The RMSD between Rhinovirus and serotype O FMDV C_α_ (excluding the G-H Loop) has previously been reported as 2.3 Å for VP1 and 1.8 Å for both VP2 and VP3 [33].

Serotypes O, A and C of FMDV share an approximate 80–82% sequence identity across the capsid proteins while the SAT-1 virus is less closely related with a sequence identity of ∼65% with the other serotypes. VP1 exhibits the most variation (∼30% for O, A and C and 50% for SAT-1) and VP2 exhibits the least variation of the surface exposed proteins (∼17% between O, A and C and 30% for SAT-1). On average the RMSD (calculated using the structural homology program [34]) of each of the surface exposed virus proteins was 0.85 Å for VP1 (excluding the G-H loop), 0.68 Å for VP2 and 0.66 Å for VP3.

### Structure Preparation

In the virus, the protein subunits are arranged with icosahedral symmetry, so the first stage in preparing the coordinates was to add the appropriate context to the selected icosahedral protomeric unit. To do this, a complete icosahedral capsid for each structure was first generated from a protomeric subunit using the 60 non-crystallographic symmetry matrices included in each coordinate file and the General Averaging Program (GAP, Stuart D.I and Grimes J.S. unpublished). From this complete structure, an icosahedral (triangular) protomeric subunit comprising VP1-4 was selected together with the protomers bounding each of the sides of the first, forming a tetrameric multimer see Figure 1(b).

The individual polypeptide chains (VP1, VP2, VP3 and VP4) that make up each protomer were relabeled [35] so each protomer was represented as a single chain, with the central protomer labeled as chain 1 and the outer protomers labeled as chains 2, 3 and 4 respectively (Figure 1(b)). This enabled the central protomer to be selected as a single chain for analysis, with all interfacing protomers being taken into account as non-selected chains where possible. Where this was not possible the entire multimer was uploaded into the epitope program and only the data for the central protomer taken forward for further analysis.

For the development of the method the surface exposure of each residue on the central protomer was determined, initially suitable criteria were established by inspection but the procedure now forms part of the software pipeline we have developed (see Files S1 and S2). In brief we use a probe the approximate size of a water molecule (1.6 Å), and any residue on the surface with greater than 1 Å^2^ exposed was included in the analysis. The interactions of residues at interfaces between protomers were determined using the PISA program [36]. Only surface exposed residues were included in the analysis.

### Identification of Picornavirus Epitopes

A literature search was conducted to collate all identified epitopes for each of the structures selected. A total of 53 and 22 neutralising epitopes were identified for Poliovirus serotype 1 [37]–[43] and Rhinovirus serotype 14 [44], [45] respectively. For each serotype of FMDV the following numbers of epitopes were identified; 25 for serotype A [30], [46]–[50], 20 for serotype O [51]–[56], 16 for serotype SAT-1 [57] and 10 for C [29], [58]–[60]. Asia-1 [61] and SAT-2 epitopes [62] were also identified, although they were not included in the analysis as there was no available capsid structure for either serotype. All of the epitopes described here, with the exception of residue 188 of VP2 which was identified using bovine mAbs, [54] are derived from murine mAbs. Only the exact residues identified were examined.

For all of the FMD structures, with the exception of the reduced O_1_ Kaufbeuren (O_1_K) structure, the immunogenic and mobile G-H loop was not represented and therefore any antigenic residues previously described on this loop were not included in the program evaluation. Additionally, as the analysis utilises structural data, only epitopes identified on the native capsid were included (i.e. monoclonal antibody escape mutants).

### Comparisons of Genetic Diversity

The genetic diversity of FMDV viruses both within serotype O and between serotypes was analysed using the Espript program [63] and a Risler similarity scoring matrix [64]. Firstly the amino acid sequences of capsids from either serotype O (N = 105) or all seven serotypes (N = 255- 105 for serotype O, 50 for serotype A, 28 for serotype Asia-1, 28 for SAT-1, 25 for SAT-2, 7 for SAT-3 and 12 for serotype C) were aligned to the amino acid sequence of the reduced serotype O1K structure file using the Clustal X program [65]. This alignment was subsequently divided into the individual proteins VP1-VP3 and any amino acid residues not found within the pdb file were removed. This meant that residues 212 and 213 of VP1 and residues 1–4 of VP2 were not included in the analysis. Each sequence file and the corresponding PDB file for each capsid protein, VP1-3, were uploaded into the program Espript.

The similarity scores generated for each residue were output in the B factor column of a new PDB file, with scores given from 100 (identical) to 0 (the most diverse). These scores were reversed so 100 represented the most diverse residues to create a ‘percentage diversity’ score.

### Determination of Program Performance

In order to evaluate the performance of each program the following parameters were determined (where ‘truth’ is defined as agreement with the experimental data described above):

*Sensitivity* = number of true positives/(number of true positives+number of false negatives)

*Specificity* = number of true negatives/(number of true negatives+number of false positives)

*Accuracy* = (true positives+ true negatives)/(true positives+number of false positives+ false negatives+true negatives)

*Precision* = true positives/(true positives+false positives)

*Probability excess* = (sensitivity+specificity) –1.

### Statistical Analysis

To determine the significance of each of the results an analysis of variance was performed using a general linear model with the virus name and the program as fixed effects. All comparisons were performed using the Tukey method for multiple comparisons, with confidence limits set at 95%. All statistical functions performed were completed using the Minitab 16 statistical software package (Minitab Inc, USA).

### Partial Automation of the Procedure for Identifying Virus Epitopes

The above procedure has been semi-automated for the use of other workers to predict picornavirus epitopes using two programs to handle the preparation (EPIPREP- file S1) and analysis of the results (EPI_PRESENT- file S2) respectively. EPIPREP takes a standard PDB file, converts the multiple chains of an icosahedral asymmetric unit to a single super-chain (providing the re-mapping information to the user), generates the full icosahedral structure from the symmetry information contained in the PDB file and selects the neighbours making the greatest contacts with the reference unit, The contacting units are numbered uniquely and an output file produced which can then be directly loaded into the epitope detection servers.

For data analysis the text files from the servers are input to EPI-PRESENT and formatted as a single merged table for the users, with residues on the inner surface of the capsid removed. Input files and a command line are also generated to facilitate graphical analysis with RIVEM [66].

Executables of EPIPREP and EPI_PRESENT are available from the authors on request and source code is provided as Supplementary Information (file S1 and S2).

## Results

### Program Optimisation

For each program in the trial, the program authors had previously established an optimal value for the threshold at which a residue is predicted to be part of an epitope by optimisation against a broad set of epitopes in a database of test structures. The metric used to set the threshold was the so-called receiver operating characteristic area under the curve (ROC AUC) value: (this is simply the area under the curve obtained by plotting true positives against false positives, both as fractions) a value greater than 0.5 represents a robust predictor performing better than chance with a value of 1 indicative of a perfect predictor. However, as this threshold is set against a broad range of epitopes it may not be appropriate for a particular system, such as virus capsids, where neotopes are likely to arise due to the juxtaposition of repeated protomer subunits.

To evaluate the utility of the suggested threshold values the performance of each of the programs was tested at 6 different threshold values against both Poliovirus and Rhinovirus. The results demonstrate that for each program a single threshold value yielded the optimal performance against both of the viruses; however, the performance between Polio and Rhinovirus is in several cases markedly different at this value (Table 1). For example, the Discotope server performed better at identifying Polio epitopes than at identifying Rhinovirus epitopes. The threshold values determined by our enterovirus based analyses were in many cases different to the default threshold suggested by the program developers, for example the Discotope server suggests a default threshold of −7.7, whereas our evaluation determined the optimal threshold to be −10.7. Additionally, the results demonstrated the generally poor sensitivity and specificity of each program tested. The picornavirus optimised threshold values were then used in the analysis of the FMDV outputs.

**Table 1:** Sensitivity and specificity results for polio and rhinovirus at the optimum threshold value for scoring a residue an epitope (the default given by the developers is also shown for comparison).

	Default server	Optimal	Poliovirus		Rhinovirus	
Program	setting	Threshold value	Sensitivity	Specificity	Sensitivity	Specificity
Discotope	−7.7	−10.7	0.868	0.746	0.591	0.686
Seppa	1.8	1.75	0.66	0.585	0.545	0.654
Epitopia	Not given	0.174	0.528	0.788	0.727	0.766
BEPro	1.3	0.8	0.717	0.767	0.682	0.612
Ellipro	0.5	0.3	0.811	0.648	0.864	0.553

### Performance of each Program at Identifying Known FMDV Epitopes

**Figure 2:**
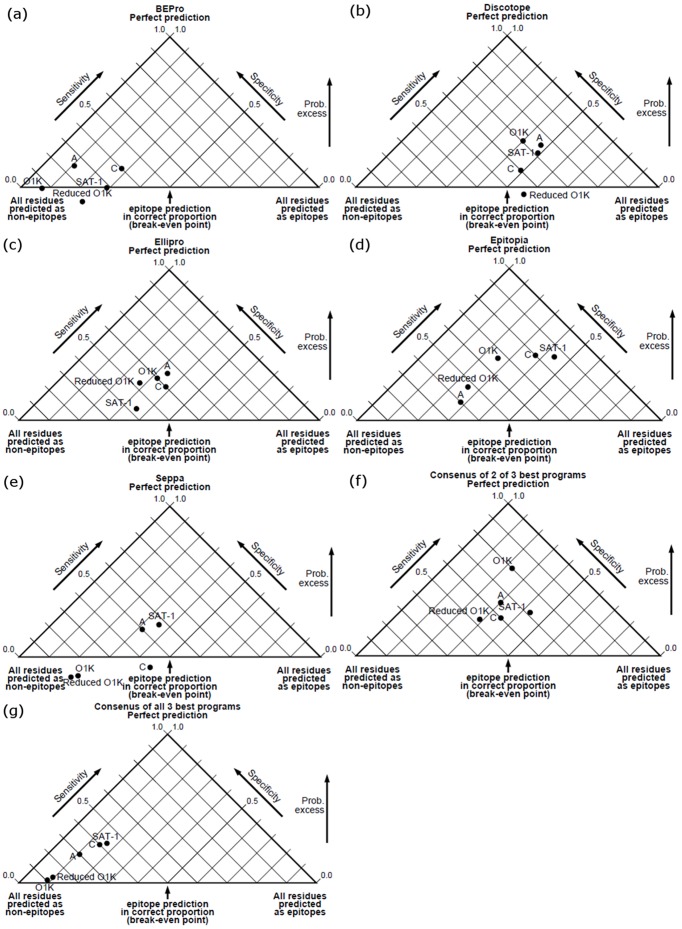
Plots of the sensitivity and specificity of each program (a–e) and a consensus of any two or all three of the top three methods at identifying the known epitopes for each FMDV structure selected represented as black dots on the pyramid plot. Pyramid probability represents scales of sensitivity and specificity, with the probability excess increasing with height and a perfect prediction (where both the sensitivity and specificity score a value of 1) coming at the tip of the pyramid. The bottom line of the plot represents the point at which results would be expected to be generated by chance.

**Figure 3:**
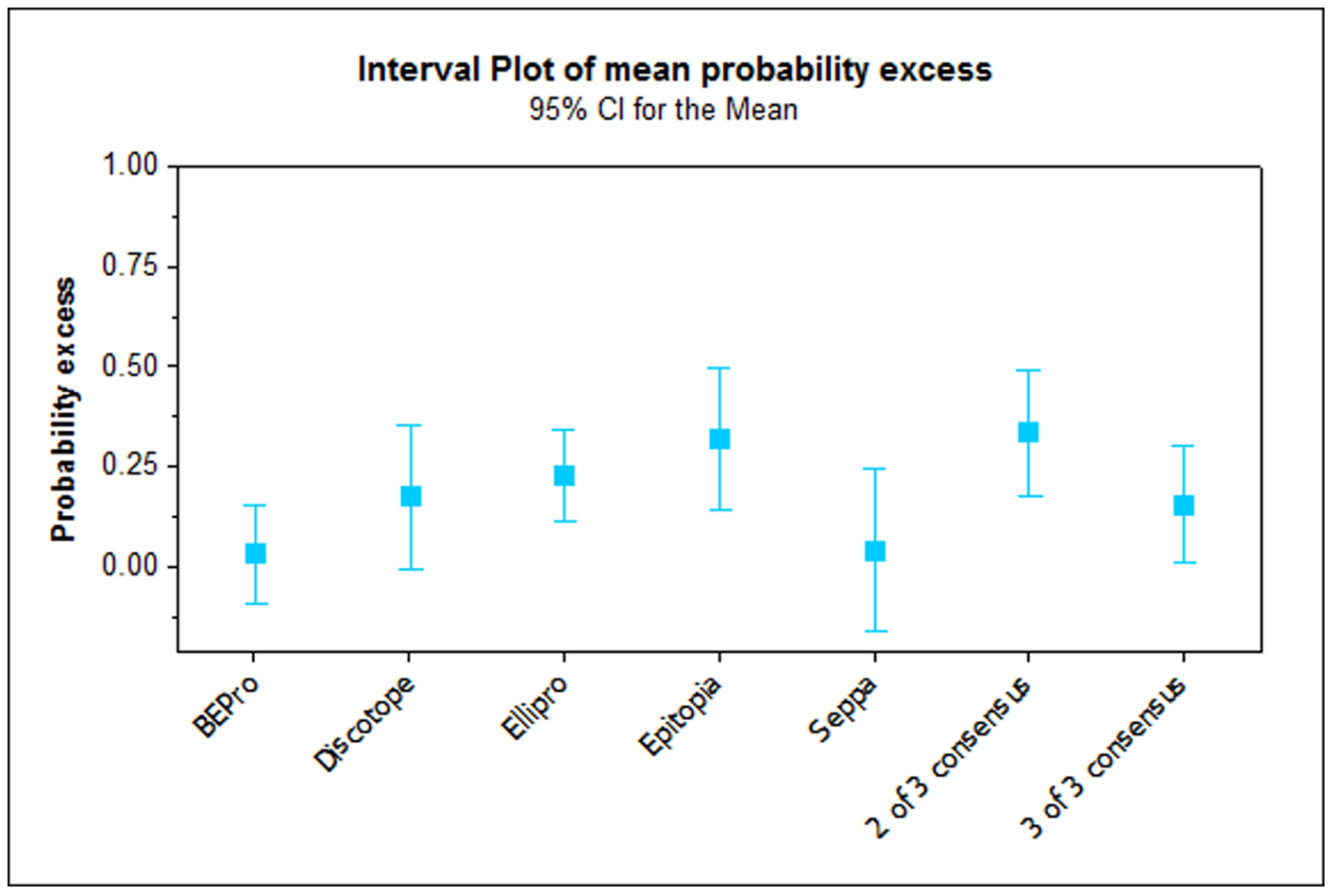
Interval plot showing the mean probability excess (and confidence intervals) of each program and a consensus of any two or all three of the top three methods.

**Figure 4:**
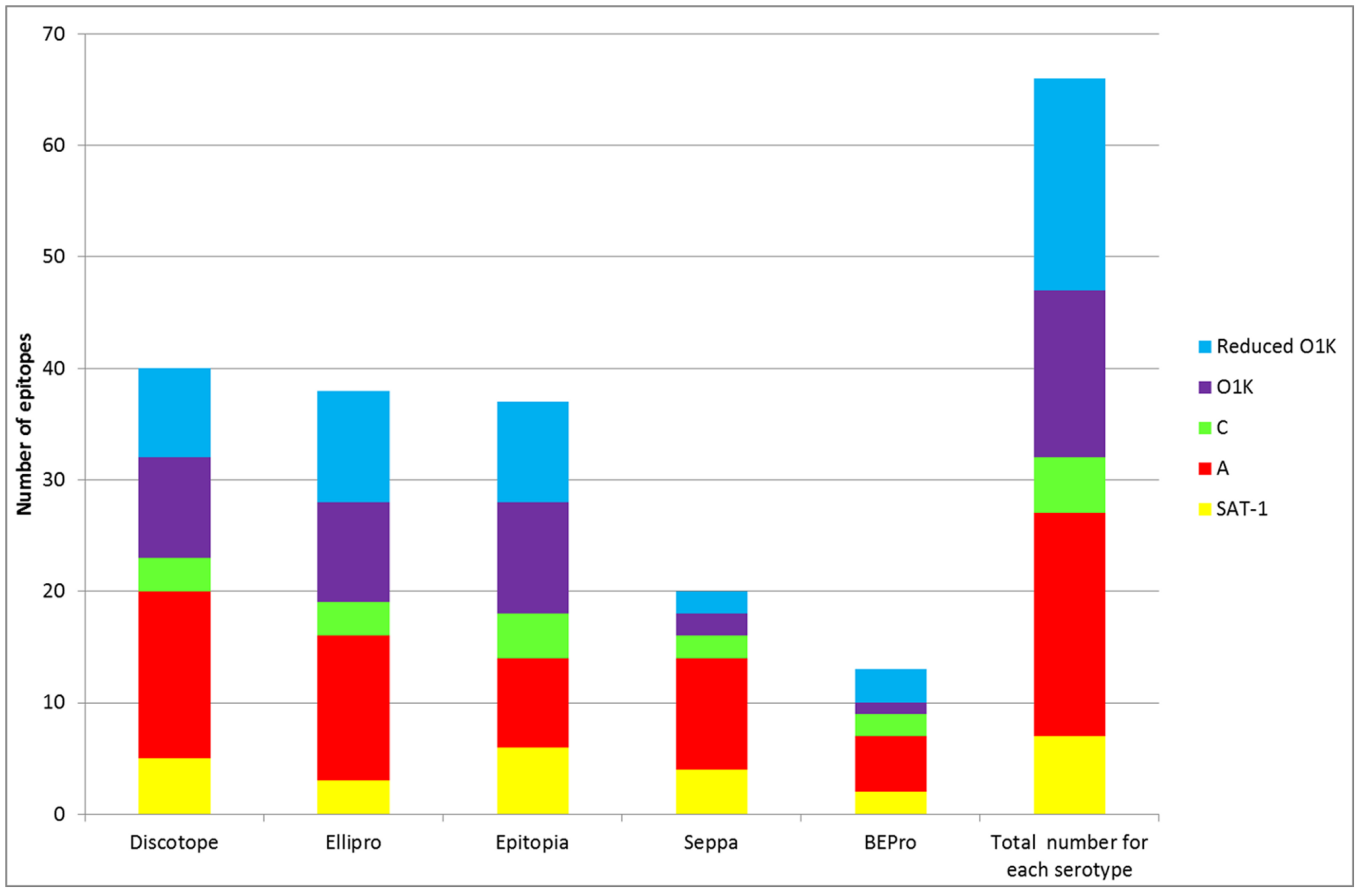
Graph showing the number of known epitopes for each serotype identified by each program compared to the total number of known epitopes for each serotype.

The best performing programs in terms of sensitivity, specificity and in the average probability excess were Epitopia and Ellipro, followed by Discotope (see Figures 2 and 3). These programs also performed well in terms of overall number of FMDV epitope residues identified (Figure 4), with Discotope correctly identifying 61% of the total of the previously described epitopes for all serotypes of FMDV, followed by Ellipro (58%) and Epitopia (56%). Seppa and BEPro performed poorly in terms of sensitivity and specificity - in some cases the programs performed worse than if the residues had been selected by chance, and average probability excess, with Epitopia performing significantly (P<0.05) better than both of these programs (Figures 2 and 3), in addition BEPro and Seppa also identified the least number of epitopes (Figure 4), with Seppa identifying 34% and BEPro 22% of the total number respectively.

**Figure 5:**
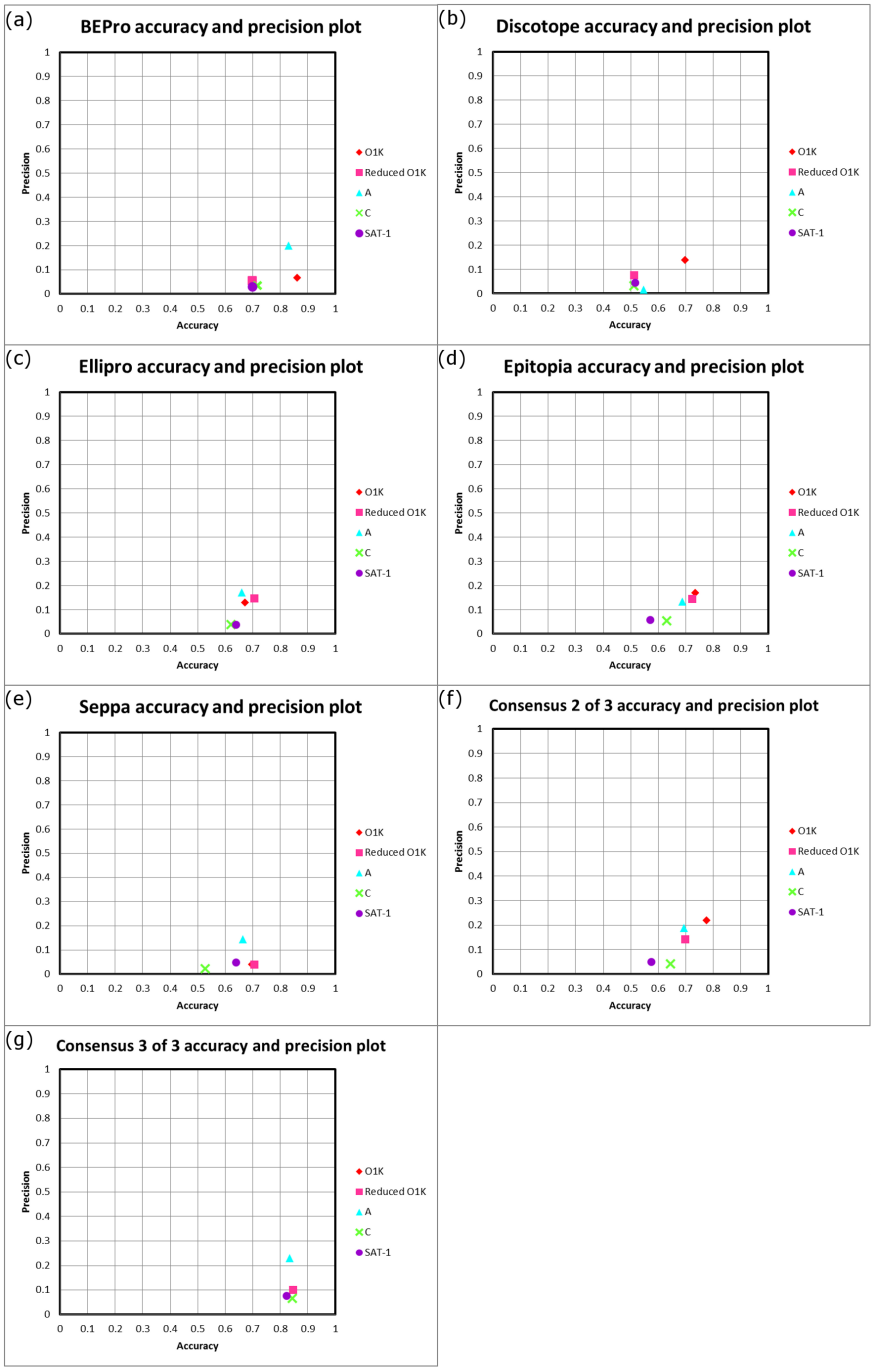
Plots of the the precision and accuracy of each program (a–e) and a consensus of any two or all three of the top three methods at identifying the known epitopes for each serotype with a structure available.

All of the programs performed poorly in terms of precision (Figure 5), with most results below 0.2. However all performed better in terms of accuracy of the results obtained, with BEPro performing the best (average 0.761), followed by Ellipro, Epitopia, Seppa and Discotope (Figure 5).

**Figure 6:**
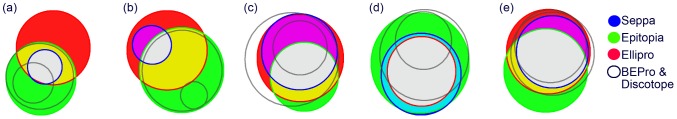
Venn diagrams showing the regognition of residues by each program for FMDV serotype O1K-Reduced (a), O1K (b), A1061 (c), SAT-1 (d) and Cs8 c1 (e). The key on the far right hand side indicates the colour of each program as represented on the Venn Diagram. For clarity the two worst perfoming algorithms are not coloured. In regions of overalap the colour is represented as the sum of the RGB colour channels of the overlapping mathods Diagrams made using the Venn master program (Kestler et al., 2008). Note that formally there need be no perfect projection of the multi-dimensional overlap information into the Venn diagram, so these represent best approximations.

Although the programs utilise different algorithms there was considerable overlap in the residues identified, and there were epitopes on each serotype that were not identified by any of the programs (Figure 4). This suggests that although the programs utilise different algorithms for identifying epitopes they are not fully independent in terms of the outputs (this is seen graphically in Figure 6).

### Using a Consensus Approach to Identify Novel Epitopes

**Figure 7:**
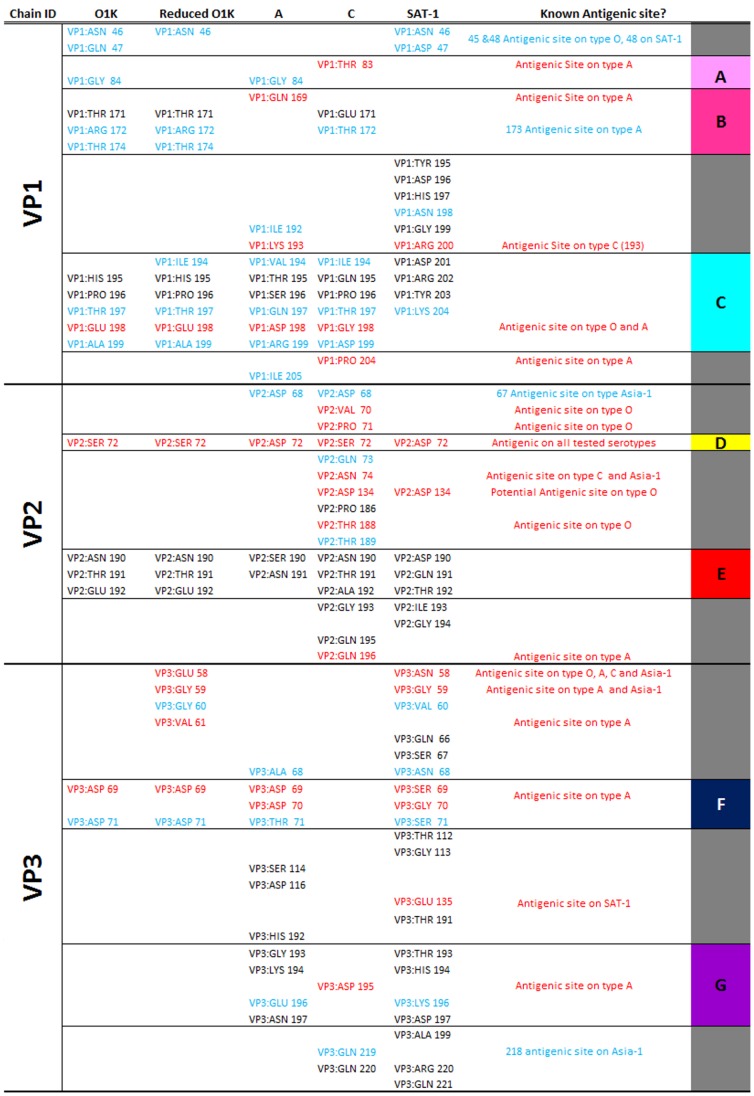
List of residues selected by a consensus of the three best performing programs (Discotope, Ellipro and Epitopia) for each selected FMDV structure compared to locations of known antigenic sites of all serotypes. Those residues coloured red are an already known epitope on at least one serotype of FMDV, those in blue are adjacent to a known epitope of FMDV. Regions A–G are predicted to be antigenic on the majority of the serotypes tested and are coloured the same on Figures 8 and 9. The remaining residues are coloured grey, as Figures 8 and 9. Note that the SAT-1 virus VP1 has a incorporated several additional residues into the G–H loop and the A serotype also aligns slightly differently to the O and C structures, therefore the numbering is different in region C as they are aligned according to position on structure. All other residues are in approximately the same location relative to each other.

To investigate the potential of using a consensus approach to predict novel epitopes the results for the three best performing programs in terms of average probability excess were selected (Epitopia, Ellipro and Discotope - see section above). The residues that were predicted to be antigenic by a consensus of all three programs on the selected structures were tabulated and compared to the known antigenic residues described previously (see Figure 7). Figure 2(g) indicates, as expected, that the sensitivity of such an approach is limited, however the significantly (P<0.01) increased accuracy obtained over any individual program (except the poorly performing program, BEPro P = 0.12) or using a consensus of 2 of the 3 programs (Figure 5(g)) validates the use of this approach. Additionally for the purpose of predicting novel epitopes using such a conservative approach is appropriate as the number of false positives should be as small as possible. It is also apparent that selecting the consensus results from any two of the best three methods gives an overall improvement in consistency, with all the sensitivity and specificity results in the right proportions (figure 2). Significant (P<0.01) improvements in the average probability excess were obtained compared to both Seppa and BEPro, and marginal, but not statistically significant, average probability excess 0.338 compared to 0.319 for the best performing program, Epitopia (Figure 2(f) and Figure 3). Whilst there was no improvement in precision or accuracy over any single program, it is possible that this approach may be useful for some applications.

**Figure 8:**
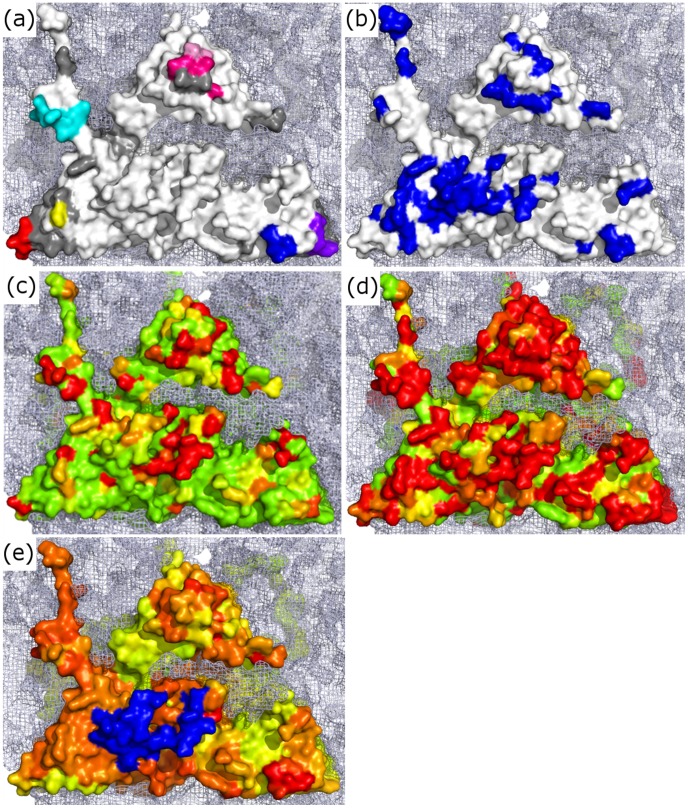
A side-by-side comparison of the molecular surface of five reduced O1 Kaufbeuren protomers made using Pymol, with the molecular surface of neighbouring protomers at each two fold axis of symmetry shown as a light blue mesh (Schrödinger LLC). Protomer (a) shows the residues in the regions A–G from Figure 7 coloured accordingly mapped onto the molecular surface (white). Protomer (b) shows the location of all described epitopes in blue for each serotype used in the analyses. Protomer (c) is a pictorial representation of the sequence variability of residues between 105 serotype O virus capsid sequences and Protomer (d) is a representation of the sequence variability of residues between 255 capsid sequences from all 7 serotypes. Both (c) and (d) were constructed using the ESPRIPT program [63] as described in the methods, the coloring goes from green (most conserved) to red (least conserved). Those sites predicted by a consensus of all programs on three or more structures tend to be located at areas of variability both inter and intra-serotypically. Region VP3 69–71 (highlighted in red on (a) and (c)) is completely conserved within serotype O, however it appears to be highly divergent between serotypes. (e) is the average RMSD across the serotypes coloured from green (smallest) to red (largest). Blue indicates the GH loop, missing on all but one structure.

**Figure 9:**
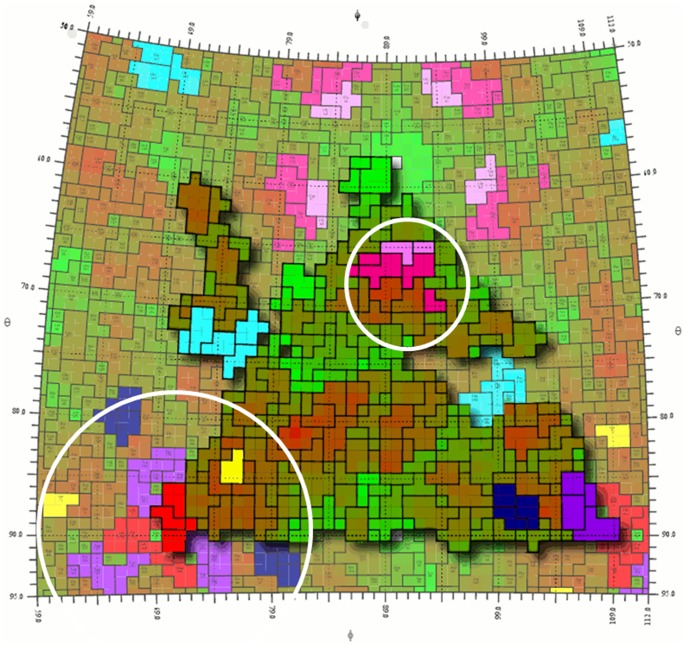
Roadmap illustration of a zoomed in area of the reduced O1K capsid generated using the RIVEM program (Xiao & Rossmann, 2007). The location of a single protomer is outlined. The occupancy of each residue on the surface is shown as an area corresponding to the surface accessibility with a black outline, with the protomer coloured according to the radius of each residue from the centre of the icosahedral capsid, coloured from dark green (least exposed) to brown (most exposed). The residues predicted by the best performing programs on the majority of the structures evaluated are coloured as in Figure 6. When the individual areas are examined it can be seen that several regions are in very close proximity across the 3-fold axis, potentially forming a conformational epitope. Additionally two of the regions are adjacent to each other near the 5-fold axis, again potentially forming a conformational epitope. These two regions are circled in white.

The majority of residues identified using this triple-consensus approach are located at regions previously described as antigenic on at least one serotype. Additionally, several of these regions were also selected for the majority (or all) of the serotypes tested (see Figure 7). This suggests that some of these residues may comprise presently unidentified epitopes on certain serotypes. The regions predicted as antigenic on the majority of serotypes are mapped onto the surface of a serotype O structure in Figures 8 and 9.

One region that is predicted for all serotypes but not previously identified as an epitope in the literature is residues 190 to 192 of VP2. This region is around the 3-fold axis and is 17 Å (from the C_α_ of the central amino acid VP2 191 to the C_α_ of VP2 72) away from residue 72 of VP2, which has previously been described as an epitope on all serotypes tested and is also predicted across all serotypes tested. This region also lies close to two other regions predicted on multiple serotypes, VP3 69–71 and 193–197 (∼25 Å and ∼14 Å from the C_α_ of the central amino acid VP2 191 to the C_α_ of VP3 70 and 195 respectively), potentially forming an antigenic site spanning the 3-fold axis across pentamers. A further area of interest lies towards the 5-fold axis of symmetry, with two regions (VP1 residues 83–84 and 169–174) identified as potentially forming a conformational epitope, again on multiple serotypes. The location of these regions is shown in Figure 9 (coloured as in Figure 7).

When the sequences of these putative epitopes are compared between different serotype O viruses (Figure 8) it can be seen that VP2 190–192 and VP1 194–199 in particular are highly variable, and so could contribute to the antigenic variation observed within this serotype. The average amino acid diversity of all surface exposed residues across the serotypes (when aligned to the reduced O1K structure) is 53.0%, this is markedly less than both the amino acid diversity of 64.5% within the predicted sites (Figure 8), and the diversity observed across known antigenic sites (67.8%). In contrast one region (VP3 69–71) is completely conserved in sequence within serotype O and so antibodies against this putative epitope might be cross-reactive against all serotype O viruses. Nevertheless even this region is not conserved across all serotypes and appears to be located on the most significant region for structural variation (Figure 8). It would appear from the variability observed between the serotypes that the prospect of finding an inter-serotypically conserved epitope is poor, at least on those parts of the capsid that are most exposed (Figure 8).

The average RMSD values (when aligned to the O_1_ Kaufbeuren structure) for the predicted sites, at 1.05 Å and for the known antigenic sites (1.13 Å) are both slightly greater than the average of the surface exposed residues (1.00 Å). For more detail see Table S1.

### The Impact of G-H Loop Conformation

The presence or absence of the G-H loop did not appear to greatly influence the results, with a similar number of epitopes identified by each program for both structures. The same regions were also predicted as being antigenic on both structures. One difference observed was the decreased sensitivity and specificity of the programs (in particular Discotope) at identifying already known epitopes on the reduced (ordered loop) form of the O_1_Kaufbeuren (Figure 2). This could be due to the inclusion of four additional epitopes located on the G-H loop that are not present on the non-reduced structure. As the G-H loop is considered to be a linear epitope these programs may not be well suited to identifying these residues.

## Discussion

A number of methods have been developed to identify epitopes, generally refined by training against globular protein/antibody complexes. The description of the methods they use is beyond the scope of this paper but they tend to analyse surface accessibility in conjunction with patch location or clustering and may include residue propensity information. The methods are therefore not entirely independent but the nature of their correlations is not fully characterised.

We have applied the programs to a slightly different problem – the detection of epitopes on the surface of virus capsids of known structure. We have developed a pipeline to allow for the complex symmetry of the virus (including a definition of the exterior surface) without needing to analyse the entire capsid. Our tests have focused on picornaviruses, and we found that the optimal threshold values of the programs for identifying both Polio and Rhinovirus epitopes differed from those identified as optimal by the program developers. This suggests that the best method for utilising these servers to predict viral epitopes is to identify the optimal threshold value for each program using a virus related closely to that being examined as a training set.

The evaluation performed in this paper has demonstrated, even after a recalibration of the methods using an appropriate training set, considerable limitations in the sensitivity and specificity of each program when applied to Picornavirus epitopes in the context of the intact capsid. Note that there may well be as yet undiscovered additional antigenic residues on the capsid, so that some predictions classified as false positives might be genuine. However any improvement in performance would be likely offset by the fact that some of the negatives classified currently as correct would probably be reclassified as false negatives.

We have found that the predictions are rendered marginally more reliable by using a consensus of any two of the best three performing programs (Figure 2(f) and figure 3). However when a consensus of all three best performing programs is taken, although the sensitivity of the results decreases, the specificity and accuracy increase significantly, indicating that this conservative approach results in less false positives and gives more confidence in the results. This is of particular importance when using the program outputs as predictions to base time consuming and expensive experiments on. We therefore examined the triple consensus results to see if there are residues which may represent undiscovered FMDV epitopes on each serotype evaluated. Several such putative epitopes were discovered and there are circumstantial reasons to believe that these predictions contain some truth. The majority of the residues predicted are located on or adjacent to a known epitope identified by monoclonal antibody techniques on at least one other serotype of FMDV. Potentially these residues are epitopes on all serotypes given the strong structural similarity of the FMDV capsids but due to the random nature of mAb selection and the discrepancy in the depth of investigation using these techniques between serotypes they may simply not have yet been discovered. Furthermore several regions are also predicted to be epitopes across serotypes. Finally the putative epitopes are on highly variable and mobile regions of the capsid which would be consistent with these regions coming under selective pressure, potentially from antibodies.

The consensus results predicted one completely novel epitope at residues 190–192 of VP2, residues which are highly variable in serotype O. Although this region is novel it should be noted that it is only slightly downstream of an epitope identified on serotype O [54]. Mapping this region to the capsid suggests that there might be a conformational epitope including both VP2 and VP3 residues spanning the 3-fold axis (and hence the pentamer-pentamer interface). A second conformational epitope is also suggested on VP1 towards the 5-fold axis at the centre of the pentamer. Thus our proof-of-principle application to FMDV has given some interesting results, which could be tested using a reverse genetics approach.

In conclusion, we believe the evaluation performed here, the first to specifically examine the outputs of these programs from the perspective of intact capsid structures, by highlighting the limited predictive power of an individual program at identifying established epitopes, suggests that it may be prudent to develop a program more specifically designed for the task of identifying viral epitopes, for example in using exclusively only epitope/paratope structures derived from viruses. However taking a triple consensus of the results from the best performing programs, suitably tuned could already provide a quick, cheap, and reasonably reliable method for predicting epitopes on several virus serotypes simultaneously. We have therefore developed a semi-automated pipeline, with executable programs available from the authors and source code provided in Supplementary Information.

## Supporting Information

Table S1 **Average diversity and RMSD of surface exposed residues, antigenic and predicted sites from all the serotypes evaluated.**
(XLSX)

File S1 **The EPIPREP source code.**
(ZIP)

File S2 **The EPI_PRESENT source code.**
(F) Click here for additional data file.
